# Identifying factors associated with intravenous fluid administration in patients with sepsis presenting to the emergency department: a retrospective cohort study

**DOI:** 10.1186/s12873-022-00650-4

**Published:** 2022-06-03

**Authors:** Gladis Kabil, Steven A. Frost, Stephen McNally, Deborah Hatcher, Aldo Saavedra, Carl J. E. Suster, Michelle Moscova, Amith Shetty

**Affiliations:** 1 School of Nursing and Midwifery, Western Sydney University, Parramatta, Australia; 2 Department of Emergency, Westmead Hospital, Westmead, Australia; 3 South Western Sydney Nursing and Midwifery Research Alliance, Ingham Institute of Applied Medical Research, Liverpool, Australia; 4 Department of Intensive Care, Liverpool Hospital and University of New South Wales, Sydney, Australia; 5 Health and Clinical Analytics, School of Public Health, The University of Sydney, Sydney, Australia; 6 Discipline of Biomedical Informatics and Digital Health, School of Medical Sciences, The University of Sydney, Sydney, Australia; 7 Faculty of Medicine and Health, University of New South Wales, Kensington, Australia; 8 Westmead Institute for Medical Research, Westmead, Australia; 9 NSW Ministry of Health, St Leonards, Australia

**Keywords:** Sepsis, Fluid therapy, Barriers, Facilitators, Early fluids

## Abstract

**Background:**

Appropriate and timely administration of intravenous fluids to patients with sepsis-induced hypotension is one of the mainstays of sepsis management in the emergency department (ED), however, fluid resuscitation remains an ongoing challenge in ED. Our study has been undertaken with two specific aims: firstly, for patients with sepsis, to identify factors associated with receiving intravenous fluids while in the ED; and, secondly to identify determinants associated with the actual time to fluid administration.

**Methods:**

We conducted a retrospective multicentre cohort study of adult ED presentations between October 2018 and May 2019 in four metropolitan hospitals in Western Sydney, Australia. Patients meeting pre-specified criteria for sepsis and septic shock and treated with antibiotics within the first 24 h of presentation were included. Multivariable models were used to identify factors associated with fluid administration in sepsis.

**Results:**

Four thousand one hundred forty-six patients met the inclusion criteria, among these 2,300 (55.5%) patients with sepsis received intravenous fluids in ED. The median time to fluid administration from the time of diagnosis of sepsis was 1.6 h (Interquartile Range (IQR) 0.5 to 3.8), and the median volume of fluids administered was 1,100 mL (IQR 750 to 2058). Factors associated with patients receiving fluids were younger age (Odds Ratio (OR) 1.05, 95% Confidence Interval (CI (1.03 to 1.07), *p* < 0.001); lower systolic blood pressure (OR 1.11, 95% CI (1.08 to 1.13), *p* < 0.001); presenting to smaller hospital (OR 1.48, 95% CI (1.25 to 1.75, *p* < 0.001) and a Clinical Rapid Response alert activated (OR 1.64, 95% CI (1.28 to 2.11), *p* < 0.001). Patients with Triage Category 1 received fluids 101.22 min earlier (95% CI (59.3 to131.2), *p* < 0.001) and those with Category 2 received fluids 43.58 min earlier (95% CI (9.6 to 63.1), *p* < 0.001) compared to patients with Triage Category 3–5. Other factors associated with receiving fluids earlier included septic shock (-49.37 min (95% CI (-86.4 to -12.4), *p* < 0.001)); each mmol/L increase in serum lactate levels (-9.0 min, 95% CI (-15.7 to -2.3), *p* < 0.001) and presenting to smaller hospitals (-74.61 min, 95% CI (-94.0 to -55.3), *p* < 0.001).

**Conclusions:**

Younger age, greater severity of sepsis, and presenting to a smaller hospital increased the probability of receiving fluids and receiving it earlier. Recognition of these factors may assist in effective implementation of sepsis management guidelines which should translate into better patient outcomes. Future studies are needed to identify other associated factors that we have not explored.

## Background

Sepsis resulting from a dysregulated immune response to infection can progress to septic shock subsequently resulting in organ failure and death [1]. It is one of the leading causes of mortality and morbidity across the world and is recognised as a global health priority [2]. Administration of early intravenous fluids to restore cardiac output remains one of the mainstays of treatment for patients with signs of sepsis-induced hypoperfusion or shock [3]. Rivers et.al [4]. in their landmark study showed a 16% reduction in the risk of mortality in patients with sepsis who received appropriate intravenous fluids within the first 6 h of presentation to the emergency department (ED).

Several recommendations have since been advocated by the Surviving Sepsis Campaign including the administration of at least 30 mL/kg of crystalloid fluids within the first 3 h; administration of intravenous antibiotics within the first hour and measurement of serum lactate [3]. A number of health care settings have adapted these guidelines and implemented locally tailored protocolised approaches to the early management of sepsis [5, 6]. Fluid administration among the suspected infection cohort of this study population has also shown mortality benefits [7]. However, adherence to these sepsis management guidelines, particularly fluid resuscitation still remains a challenge in the ED [8–10] warranting an exploration of the factors specifically associated with fluid administration. An understanding of these factors is essential to design and implement tailored performance improvement initiatives targeting fluid administration rather than a “one size fits” all approach. Any improvement in sepsis care will reduce sepsis related mortality and morbidity.Therefore, this study was undertaken with two specific aims: firstly, for patients with sepsis, to identify factors associated with receiving intravenous fluids while in the ED; and, secondly among patients who receive fluids to identify determinants associated with actual time to fluid administration.

## Methods

### Study setting

We conducted a multicentre retrospective observational study as part of the Sydney Multicentre Emergency Department Sepsis Archive from four EDs in Western Sydney, Australia with a combined 200,000 ED visits per year. Two of these were larger tertiary level hospitals and two of them were smaller secondary level hospitals. The study period for this project was between October 2018 and May 2019, after the introduction of an electronic fluid management record at each site. All the participating EDs used the Sepsis Pathway as part of the Sepsis Kills Program [11] with recommendation to commence the fluids immediately on recognising sepsis. Ethical approval for the study was obtained from the Western Sydney Local Health District Human Research Ethics Committee (HREC2014/3/5.3(3939) AU RED LNR/14/WMED/66) and permission to access the electronic medical record data was obtained from the appropriate data custodians. The STROBE guidelines have been used to report the results of this study [12].

### Study population and data source

Data included encounters from all adult presentations (over 16 years of age) who presented to the participating EDs. Sepsis was defined as those who met the suspected infection criteria for Sepsis 3 definition [1] with an initial modified Sequential Organ Failure Assessment (mSOFA) ≥ 2 and antibiotics administered within the first 24 h. The modified ED-based mSOFA score was derived using an approach reported by Shetty et al. [13]. Septic shock was defined as sepsis with a systolic blood pressure < 90 mmHg and a lactate of ≥ 2 mmol/L. Patients were considered to have received fluids if they received intravenous fluid in the emergency department within the first 24 h. The time to fluid administration was calculated from the time of diagnosis of sepsis to the initiation of first intravenous fluid. The time of diagnosis of sepsis was determined by the time of the vital signs used for the worst mSOFA score within the first three hours of encounter. The time and the volume of fluid given, along with charactersistics of the study participants and their presentation to the emergency department were obtained from the electronic Medical Record (eMR).This data was extracted by two experienced data scientists. The Charlson Comorbidity Score [14] was derived from the Snowmed codes used in the eMR. The Clinical Rapid Response Alert refers to the Red Zones representing late warning signs of deterioration for vital signs observations as defined by the Clinical Excellence Commission Adult Sepsis Pathway [11] used in the study hospitals. The values are the number of times the vital signs exceeded this threshold.

### Statistical analysis

All analysis was performed using R statistical language software (version 4.0.3, R Foundation for Statistical Computing, Vienna, Austria). Descriptive summaries were produced for: (1) all presentations with sepsis; (2) the subset of these patients who received intravenous fluids; (3) patients who did not receive intravenous fluids; and (4) patients receiving intravenous fluids, categorised by the time of first intravenous fluid administration (< 1 h, 1- < 3 h, 3- < 6 h and 6- < 24 h). Multivariable logistic regression models were used to identify factors associated with receiving intravenous fluid, both crude (univariate) and adjusted estimates of effect are presented as Odds Ratios (OR), 95% confidence intervals (95% CI), and only *p*-values for final models are presented. All potential covariates were included in the initial model, and the final model was developed using a stepwise (combined forward and backward elimination, with a *p*-value threshold of 0.20 for retention of potential covariates) approach, and the overall model fit was assessed using Akaike Information Criterion (AIC), the Area Under the Receiver Operator Curve (AUC) for the adjusted and final models which are also presented [15]. To explore the factors associated with time to receiving intravenous fluids, a multivariable linear regression model was used to estimate the association between factors and the average time to intravenous fluid administration. The final linear regression model was fitted using a similar stepwise approach. All potential pairwise-interaction effects were initially examined, using a *p*-value of 0.10 as a cut-off, no interaction terms were identified as significant. For these linear multivariable models, the overall fit of the model was assessed using an adjusted R^2^.

## Results

**Fig. 1:**
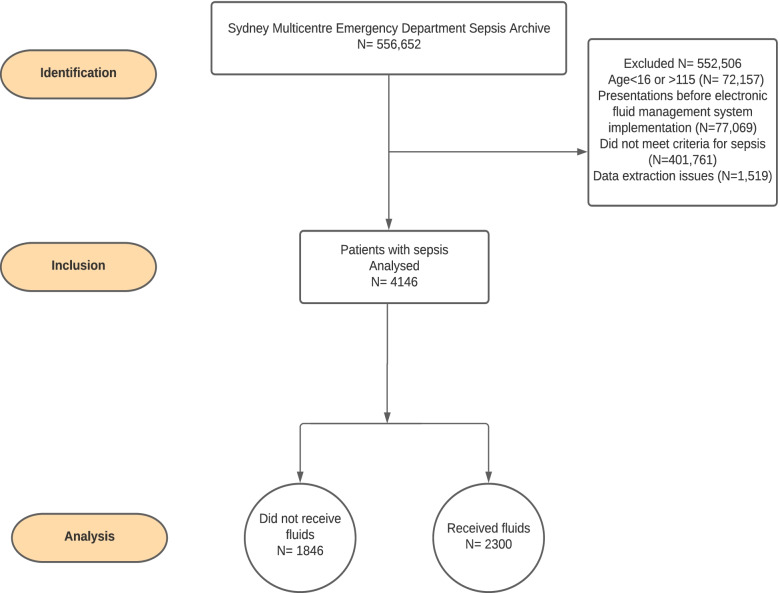
Flow-diagram of included participants

From the initial 556,652 ED presentation records, 4146 patients met the inclusion criteria for sepsis (Fig. 1). Of these, 2,300 (55.5%) patients received intravenous fluids and 1,846 (44.5%) patients did not receive fluids. Among those who received fluids, the median time to first intravenous fluid administration was 1.6 h (Interquartile Range (IQR) 0.5 to 3.8), and the median volume of intravenous fluids administered was 1100 mL (IQR 750 to 2058). The median time to completion of the first intravenous fluids was 5.8 h (IQR 2.8 to 10.6).

Characteristics of patients who received fluids and did not receive fluids are presented in Table 1. For example, sepsis patients who received fluids in the ED were on average younger (median of 73-years versus 75-years, respectively, *p* < 0.001). A similar distribution of women and men who did and did-not receive fluids (*p* = 0.60) was found. Patients receiving fluids weighed less (72 kg versus 75 kg, *p* < 0.001), and had a similar number of documented comorbidities (Charlson Comorbidity Score, mean (SD) 0.60 (1.19) and 0.76 (1.40) respectively, *p* = 0.005). Patient subgroup receiving fluids had a greater proportion of Triage Category 1 ( Immediate Simultaneous Assessment and Treatment) [16] and Category 2 (Assessment and treatment within 10 min) patients (*p* < 0.001) compared to Triage Category 3 ( Assessment and treatment start within 30 min); Category 4 (Assessment and treatment start within 60 min); and Category 5 (Assessment and treatment start within 120 min), a higher mSOFA (median of 3 versus 2, respectively, *p* < 0.001), higher median lactate and lower median systolic blood pressure (both *p*-values < 0.001). Subgroup receiving fluids had significantly higher proportion of septic shock patients (10% versus 2%, septic and non-septic shock patients, *p* < 0.001). Sepsis patients presenting to a smaller compared to larger hospital EDs were more likely to receive fluids (61% versus 53%, *p* < 0.001). Similarly, patient characteristics based on time to fluid administration categories are presented in Table 2. Patients who received intravenous fluids earlier received larger volume of fluids.

**Table 1:** Comparison of patient characteristics among those with sepsis based on intravenous fluids received vs not received within the first 24 h in the emergency department (*N* = 4146)

Group	N	All Patients (*n* = 4146)	Received fluids (*n* = 2300)	Did not receive fluids (*n* = 1846)	*P*-value
Age, median (IQR) years	4146	74 (61 to 83)	73 (58 to 83)	75 (63 to 84)	< 0.001
Men N (%)	4146	2227 (54)	1227 (53)	1000 (54)	0.600
Weight, median (IQR), kg	2648	74 (61 to 90)	72 (60 to 89)	75 (63 to 92)	< 0.001
Charlson Comorbidity Score Mean (SD)	3941	0.67 (1.29)	0.60 (1.19)	0.76 (1.40)	0.005
Triage Category N (%)	4146				
1		252 (6)	172 (7)	80 (4)	< 0.001
2		2030 (49)	1205 (52)	825 (45)	< 0.001
3–5		1864 (45)	923 (40)	941 (51)	< 0.001
Presentation Hour N (%)	4146				
0700-1500 h		2091 (50)	1129 (49)	962 (52)	1.000
1500-2300 h		1536 (37)	891 (39)	645 (35)	0.753
2300-0700 h		519 (13)	280 (12)	239 (13)	< 0.001
Modified Sofa Score (mSOFA), Median (IQR)	4146	3.0 (2.0 to 4.0)	3.0 (2.0 to 4.0)	2.0 (2.0 to 4.0)	< 0.001
Lactate, Median (IQR), mmol/L	3255	1.7 (1.2 to 2.4)	1.8 (1.3 to 2.7)	1.5 (1.1 to 2.1)	< 0.001
Lowest SBP, Median (IQR), mmHg	4135	107 (97 to 122)	105 (92 to 116)	114 (103 to 127)	< 0.001
Septic Shock N (%)	4090				
Yes		258 (6)	228 (10)	30 (2)	< 0.001
No		3832 (94)	2035 (90)	1797 (98)	< 0.001
Clinical Rapid Response Alert Activated N (%)	4146				
0		3491 (84)	1805 (78)	1686 (91)	< 0.001
1		524 (13)	393 (17)	131 (7)	< 0.001
2		115 (3)	89 (4)	26 (1)	< 0.001
3		16 (0)	13 (1)	3 (0)	< 0.001
On Immunosuppressants N (%)	4146				
Yes		28 (1)	10 (0)	18 (1)	< 0.001
No		4118 (99)	2290 (100)	1828 (99)	< 0.001
On Steroids N (%)	4146				
Yes		68 (2)	34 (1)	34 (2)	0.360
No		4078 (98)	2266 (99)	1812 (98)	0.281
Antibiotic administration time, Median (IQR), hours	4146	4.25 (2.3 to 8.7)	2.3 (0.7 to 5.2)	3.5 (1.6 to 7.3)	< 0.001
Hospital Site N (%)	4146				
Larger Hospitals		2871 (69)	1527 (66)	1344 (73)	< 0.001
Smaller Hospitals		1275 (31)	773 (34)	502 (27)	< 0.001

**Table 2:** Comparison of patient characteristics among those with sepsis based on sepsis diagnosis to intravenous fluid administration time within the first 24 h in the emergency department (*N* = 2300)

	**Sepsis Diagnosis to fluid administration time (hours), *N* = 2300**				
**Group**	** < 1 h** **(*n* = 856)**	**1- < 3 h** **(*n* = 702)**	**3- < 6 h** **(*n* = 442)**	**6- < 24 h** **(*n* = 300)**	***P*-value**
Age, median (IQR) years	70 (52 to 82)	73 (57 to 83)	77 (63 to 85)	76 (66 to 85)	< 0.001
Men N (%)	470 (55)	365 (52)	232 (52)	160 (53)	0.690
Weight, median (IQR), kg	72 (60 to 89)	72 (60 to 88)	72 (62 to 86)	75 (61 to 91)	0.340
Charlson Comorbidity Score Mean (SD)	0.52 (1.12)	0.56 (1.11)	0.71 (1.27)	0.80 (1.42)	0.003
Triage Category N (%)					
1	108 (13)	36 (5)	16 (4)	12 (4)	< 0.001
2	500 (58)	367 (52)	198 (45)	140 (47)	< 0.001
3–5	248 (29)	299 (43)	228 (52)	148 (49)	< 0.001
Presentation Hour N (%)					
0700-1500 h	437 (51)	321 (46)	226 (51)	145 (48)	0.002
1500-2300 h	302 (35)	301 (43)	173 (39)	115 (38)	0.001
2300-0700 h	117 (14)	80 (11)	43 (10)	40 (13)	< 0.001
mSOFA score, Median (IQR)	3.0 (2.0 to 4.0)	3.0 (2.0 to 4.0)	3.0 (2.0 to 4.0)	3.0 (2.0 to 4.0)	0.002
Lactate, Median (IQR), mmol/L	2.0 (1.4 to 3.0)	1.8 (1.3 to 2.6)	1.6 (1.2 to 2.3)	1.7 (1.2 to 2.4)	< 0.001
Lowest SBP, Median (IQR), mmHg	99 (87 to 111)	103 (93 to 115)	105 (95 to 119)	106 (98 to 123)	< 0.001
Septic Shock N (%)					
Yes	131 (16)	65 (9)	22 (5)	10 (3)	< 0.001
No	712 (84)	629 (91)	408 (95)	286 (97)	< 0.001
ICU Admission N (%)					
Yes	90 (11)	66 (9)	22 (5)	25 (8)	< 0.001
No	766 (89)	636 (91)	420 (95)	275 (92)	0.009
Clinical Rapid Response Alert Activated N (%)					
0	625 (73)	565 (80)	362 (82)	253 (84)	0.053
1	175 (20)	107 (15)	68 (15)	43 (14)	0.152
2	46 (5)	28 (4)	11 (2)	4 (1)	< 0.001
3	10 (1)	2 (0)	1 (0)	0 (0)	< 0.001
On Immunosuppressants N (%)					
Yes	2 (0)	7 (1)	1 (0)	0 (0)	0.053
No	854 (100)	695 (99)	441 (100)	300 (100)	0.800
On Steroids N (%)					
Yes	9 (1)	12 (2)	8 (2)	5 (2)	0.630
No	847 (99)	690 (98)	434 (98)	295 (98)	0.783
Antibiotic administration time, Median (IQR), hours	0.20 (0.0 to 1.0)	0.85 (0.1 to 2.7)	1.75 (0.6 to 3.7)	3.35 (1.4 to 7.25)	< 0.001
Total Volume of fluids administered within first 24 h, Median (IQR), mL	1750 (1000 to 2952)	1250 (976 to 2138)	1000 (530 to 1800)	735 (320 to 1016)	< 0.001
Hospital Site N (%)					
Larger Hospitals	505 (59)	465 (66)	311 (70)	246 (82)	< 0.001
Smaller Hospitals	351 (41)	237 (34)	131 (30)	54 (18)	< 0.001

### Receiving fluids

The factors associated with patients with sepsis receiving intravenous fluids compared to not receiving fluids are presented in Table 3. Factors associated with receiving fluids included: younger age, (Odds Ratio (OR) for each 5-year decrease in age = 1.05, 95% Confidence Interval (CI) (1.03 to 1.07), *p* < 0.001); lower systolic blood pressure (OR for each 5 mmHg decrease = 1.11, 95% CI (1.08 to 1.13), *p* < 0.001); presenting to a smaller compared to a larger hospital ED (OR = 1.48, 95% CI 1.25 to 1.75, *p* < 0.001); and a Clinical Rapid Response alert activated (OR 1.64, 95% CI (1.28 to 2.11), *p* < 0.001).

**Table 3:** Factors associated with receiving intravenous fluids among sepsis patients presenting to the emergency department

		**Received fluids vs Not received fluids**			
	**Units of comparison**	**Crude OR (95% CI)**	**Adjusted OR (95% CI)**	**Final Model Adjusted OR (95% CI)**	***P* value**^**1**^
Age	5-year decrease	1.05 (1.03 to 1.08)	1.05 (1.03 to 1.07)	1.05 (1.03 to 1.07)	< 0.001
Men	vs Women	0.96 (0.83 to 1.11)	0.93 (0.79 to 1.08)		
*Triage Category*					
3–5		1.0 (Ref)	1.0 (Ref)	1.0 (Ref)	
2		1.31 (1.12 to 1.52)	1.09 (0.92 to 1.28)	1.11 (0.94 to 1.3)	0.240
1		1.62 (1.2 to 2.18)	1.23 (0.87 to 1.73)	1.22 (0.87 to 1.71)	0.217
mSOFA score	1- unit increase	1.12 (1.06 to 1.18)	0.94 (0.87 to 1.02)	1.11 (0.99 to 1.18)	0.172
Lactate	1 mmol/L increase	1.21 (1.14 to 1.29)	1.07 (1.0 to 1.14)	1.05 (0.98 to 1.12)	0.179
Charlson Comorbidity Score	1-unit increase	0.94 (0.89 to 1.0)	0.97 (0.92 to 1.03)	0.91 (0.82 to 1.01)	0.216
Septic shock Yes	vs No	5.31 (3.6 to 7.82)	1.94 (1.25 to 3.0)	1.83 (1.15 to 2.9)	0.011
*ICU admission* Yes	vs No	01.26 (0.98 to 1.61)	0.92 (0.69 to 1.23)		
*Presentation hour*					
0700-1500 h		1.0 (Ref)	1.0 (Ref)		
1500-2300 h		1.14 (0.96 to 1.35)	1.13 (0.94 to 1.35)		
2300-0700 h		0.99 (0.78 to 1.26)	0.97 (0.75 to 1.26)		
Lowest Systolic BP	5 mmHg decrease	1.14 (1.12 to 1.17)	1.1 (1.09 to 1.13)	1.11 (1.08 to 1.13)	< 0.001
*On Immunosuppressants* Yes	vs No	0.44 (0.19 to 1.04)	0.4 (0.15 to 1.08)	0.38 (0.15 to 0.96)	0.037
*On Steroids* Yes	vs No	0.87 (0.51 to 1.49)	1.02 (0.54 to 1.95)		
Antibiotic administration time	1-h increase	0.99 (0.98 to 0.99)	0.99 (0.98 to 1.0)	0.99 (0.98 to 1.00)	0.007
*Hospital Sites* Smaller Hospitals	vs larger hospitals	1.28 (1.09 to 1.49)	1.35 (1.14 to 1.6)	1.48 (1.25 to 1.75)	< 0.001
Clinical Rapid Response Alert Activated	1- unit increase	2.41 (1.97to 2.95)	1.54 (1.2 to 1.98)	1.64 (1.28 to 2.11)	< 0.001
AUC				0.695	
*N* = 3094					

^**1**^*P* values from the final model

### Time to fluids

Several factors were found to be associated with the time to intravenous fluids. Triage Category 1 and 2 were associated with receiving fluids 101.22 min earlier (95% CI (59.3 to 131.2), *p* < 0.001) and 43.58 min earlier (95% CI (9.6 to 63.1), *p* < 0.001) respectively compared with those triaged as a less urgent Triage Category 3, 4 or 5. Patients with septic shock received intravenous fluids 49.37 min earlier (95% CI (12.4 to 86.4), *p* < 0.001) compared with those with no signs of septic shock. Each one mmol/L increase in serum lactate level (-9.0 min, 95% CI (-15.7 to -2.3), *p* < 0.001) showed statistically significant association with reduced time to fluids. Patients presenting to smaller secondary hospitals received their first intravenous fluids 74.61 min earlier (95% CI (55.3 to 94.0), *p* < 0.001) than those who presented to larger tertiary hospitals. Factors such as documented immunosuppressant therapy at the time of presentation (-85.02 min; 95% CI (-213.9 to 43.9), *p* = 0.186) and mSOFA score, for each unit increase (5.9 min; 95% CI (-0.69 to 13.5), *p* = 0.524) showed no association with reduction in time to fluids. While these factors were associated with early administration of intravenous fluids, some factors were associated with delayed fluid administration.

Each 5-year increase in age was associated with a 4.9 min delay in receiving fluids (95% CI (2.5 to 7.3), *p* < 0.001). Other factors associated with delays in receiving fluids included the systolic blood pressure with each 5 mmHg increase being associated with 15.13 min delay (95% CR (1.45 to 28.81), *p* < 0.001). Each hour increase in the time to intravenous antibiotics administration was associated with 2.25 min fluid delay (95% CI (1.4 to 3.1), *p* < 0.001) (Table 4).

**Table 4:** Determinants of time (in minutes) to intravenous fluid administration in patients with sepsis in the emergency department

		**Triage to fluid administration time (min)**			
	**Units of comparison**	**Crude Co-eff (mins) (95% CI)**	**Adjusted Co-eff (mins) (95% CI)**	**Final Model Adjusted Co-eff (mins) (95% CI)**	***P* value**^**1**^
Age	5-year increase	5.74 (3.3 to 8.19)	5.17 (2.72 to 7.62)	4.9 (2.5 to 7.3)	< 0.001
Men	vs Women	0.88 (-19.5 to 21.3)	11.94 (-8.1 to 31.9)		
*Triage Category*					
3–5		1.0 (Ref)	1.0 (Ref)	1.0 (Ref)	
1		-105.79 (-143.2 to -68.3)	-101.71 (-141.1 to -62.3)	-101.22 (-131.2 to-59.3)	< 0.001
2		-50.02 (-69.6 to -30.4)	-42.59 (-62.6 to -22.6)	-43.58 (-63.1 to-9.6)	< 0.001
mSOFA score	1- unit increase	-3.66 (-9.8 to -2.4)	3.15 (-5.75 to 12.1)	5.9 (-0.69 to13.5)	0.524
Lactate	1 mmol/L increase	-14.11 (-19.6 to -8.7)	-8.15 (-14.8 to -1.9)	-9.0 (-15.7 to -2.3)	< 0.001
Charlson Comorbidity Score	1-unit increase	8.88 (0.83 to 16.92)	4.59 (-2.93 to 12.7)		
Septic Shock Yes	Vs No	-83.21 (-112.7 to -54.4)	-41.04 (-75.4 to -6.6)	-49.37 (-86.4 to -12.4)	< 0.001
*ICU admission* Yes		-10.99 (-42.5 to 20.5)	10.29 (-22.4 to -43.0)	0.69 (-31.6 to -33.0)	0.159
*Presentation hour*					
0700-1500 h		1.0 (Ref)	1.0 (Ref)	1.0 (Ref)	
1500-2300 h		15.01 (-6.8 to 36.9)	19.53 (-1.4 to 40.5)		
2300-0700 h		0.02 (-31.8 to 31.9)	7.59 (-23.0 to 38.1)		
Lowest Systolic BP	5-mm Hg increase	32.21 (20.07 to 44.35)	12.86 (-1.17 to 26.89)	15.13 (1.45 to 28.81)	0.001
*On Immunosuppressants* Yes	vs No	-59.47 (-205.3 to 86.4)	-128.1 (-278.9 to 22.7)	-85.02 (-213.9 to 43.9)	0.186
*On Steroids* Yes	vs No	53.03 (-21.6 to 127.6)	63.24 (-14.1 to 140.6)		
Antibiotic administration time	1-h increase	3.04 (2.2 to 3.9)	2.14 (1.3 to 3.0)	2.25 (1.4 to 3.1)	< 0.001
*Hospital Sites* Smaller Hospitals	vs larger hospitals	-58.85 (-78.2 to -39.5)	-68.56 (-88.2 to -48.9)	-74.61 (-94.0 to -55.3)	< 0.001
Clinical Rapid Response Alert Activated	1- unit increase	-41.03 (-62.6 to -19.5)	-3.26 (-30.8 to 24.3)	3.05 (-21.5 to 27.6)	0.808
R Squared				0.10	
*N* = 1946					

^**1**^*P* values from the final model

## Discussion

Our study has found that on average just over half of the patients presenting to the ED with sepsis received fluids which is less than optimal, and the average time to first fluids was 1.6 h. Unlike other elements of the Surviving Sepsis Campaign bundle, studies have scarcely explored factors associated with intravenous fluid administration [10]. Our study has identified a number of factors associated with initiation of intravenous fluids in patients with sepsis in the ED. Being younger age, lower triage category and having more severe clinical signs of sepsis increased the probability of receiving fluids and reduced the time to first fluids. The only non-patient factor related to variation in the probability of receiving fluid was presentation to a smaller hospital. Recognition of these factors may assist in developing and effectively implementing tailored performance improvement initiatives. Given the high incidence and mortality rates related to sepsis, any improvement in sepsis care can translate to substantial benefit to patients.

The proportion of patients who received fluids in this study is similar to previous retrospective studies [9, 17]. The average time to first fluid administration was also similar compared to other studies [18–21] conducted in settings that use local protocols adapted from the Surviving Sepsis Campaign guidelines [22] similar to the Sepsis Pathway [11] used in the participating hospitals in this study.

On exploring the factors associated with receiving versus not receiving intravenous fluids, patients who were younger were more likely to receive fluids and increasing age was associated with delayed intravenous fluid administration. This finding is similar to previous studies reporting clinicians’ hesitancy to prescribe fluids in the older population [23] despite evidence supporting significance of fluids in the older people with sepsis to improve preload and mortality while using a cautious approach [24, 25].

In line with our expectations, a higher proportion of patients with more severe clinical signs of sepsis received intravenous fluids and they were also more likely to receive fluids early. This is most likely associated with indication bias where sicker patients were more likely to be recognised and treated earlier. Recognisable features of illness severity that showed association in this study include signs of septic shock, low systolic blood pressure, and increasing lactate level. Patients who were on immunosuppressants who were more likely to develop severe infections also received fluids earlier. These findings suggest the possibility that clinicians predominantly rely on clinical judgement while administering fluids. While the importance of clinical judgement cannot be disputed, the fact that clinician factors such as inexperience, failure to recognise sepsis, clinical reliance on development of explicit signs of hypotension that have been reported previously as barriers to timely intravenous fluid initiation in sepsis cannot be ignored [9, 17, 26]. In addition, change to SOFA definition [13] could mean that patients may meet sepsis definitions without being hypotensive or showing signs of volume depletion and only receive fluids when they present those symptoms and therefore sicker. Future studies will need to focus on whether fluid resuscitation is important in both groups of sepsis – sepsis with or without hypotension.

Nearly half of the patients with sepsis in this study did not receive intravenous fluids in the ED within the first 24 h. This finding should be interpreted with caution as the study is subject to the limitations of retrospective analysis of information retrieved from eMR and we are unable to associate individual factors such as “Not for Resuscitation” orders, fluid restriction status and any other relevant clinical indications that might potentially contribute to patients not receiving fluids. Nevertheless, the significant proportion of patients who meet the criteria for sepsis or septic shock yet not receiving fluids warrant further exploration in future studies.

While among those who received fluids, a total of about 68% of patients received fluids within the first three hours, the others received fluids later. While administration of intravenous fluids within the first 30 min [27] and larger volume resuscitation [7] has been shown to have mortality benefits, some studies report potential harm related to injudicious fluid administration [28, 29]. In addition, ongoing controversies regarding the benefit of volume resuscitation might contribute to clinical indecision with recent clinical trials failing to demonstrate mortality benefits [30–32] as shown in the Rivers et.al. study [4]. However, it is necessary to note that the studies advocating a conservative approach do not contradict the need for early administration of initial intravenous fluids. They rather warn against unwarranted cumulative fluid volume overload beyond the early resuscitation phase. This approach is in line with the current Surviving Sepsis guidelines.

Contrary to our expectations and previous study findings [8], other patient-related factors such as co-morbidities did not show association with receiving fluids or time to fluids in our adjusted models. This may be due to the low number of patients in this study with documented pre-existing co-morbidities. The time of presentation showed no association with the likelihood of receiving fluids or the time to fluids. The plausible explanation for this could be that all participating hospitals were in metropolitan regions with availability of medical and nursing personnel throughout the day. Similarly, time to antibiotic administration did not show significant association with receiving intravenous fluids. This is in contrast to the perception that antibiotic administration takes precedence over fluids [33].

The only non-patient related factor that showed significant association with both the likelihood of receiving fluids and receiving fluids early was presentation to smaller secondary hospitals. While this may be attributed to the characteristics of the tertiary settings which are typically overcrowded resulting in delays in fluid resuscitation [7, 8], it is also possible that other organisational factors related to model of care could be contributing to the difference. For instance, at the smaller hospitals in this study, all patients with Triage Category 1 and Category 2 were cared for in the Resuscitation Bays with a nurse-to-patient ratio of 1:1 or 1:2. In contrast, at the larger hospitals, patients with a Triage Category 2 could be cared for in Acute Care Bays with a nurse-to-patient ratio of 1:4 or 1:5.

The estimates obtained from our modelling of the timing of fluid administration appear to make clinical sense. For example, using the results from the final model to predict the time to fluid, it was estimated that a young adult patient presenting to a participating smaller ED with sepsis triaged as a Category 2, and a systolic blood pressure of 90 mmHg, lactate more than 2 mmol/L is predicted to receive intravenous fluids 2.4 h earlier than a patient who does not have these characteristics. Recognising these factors influencing fluid administration can be of relevance to clinical practice.

The results of this study need to be considered in the context of some potential weaknesses. Despite the large database allowing relevant inference about clinical practice, this was a retrospective observational study and therefore, there is a possibility of unaccounted confounders and causality cannot be established. Although our study included four different hospitals within a single local health district, the findings may not reflect the situation of all EDs in Australia. Some data was missing due to incomplete or data entry errors in the eMR which could potentially have an inclusion bias. Some potentially relevant information such as experience level of the clinician, time of first contact with the medical officer, time of first contact with the nurse after triage etc. could not be retrieved. In addition, the Charlson Co-morbidity Score used in this study was derived using data from admission records, and any detail recorded in medical assessment could not be retrieved potentially impacting the accuracy of the calculated score. The eMR may not have included all interventions performed, for instance information regarding pre-hospital fluid administration were not available. Furthermore, there was a lack of information about other potential causes of hypotension. However, the data extraction process with the electronic time stamps allowed recovery of the majority of potentially relevant variables.

Importantly, even though our study has identified several factors associated with who and when individuals presenting with sepsis received fluids in ED, there may be a number of factors we may have missed. They may be factors not routinely captured in patient records such as organisational culture, perceptions and knowledge related issues. Further investigation of other associated factors using qualitative and mixed approaches is essential to obtain a deeper understanding of the facilitators and barriers.

## Conclusion

Our study has found that on average just over half of patients presenting to the ED with sepsis received fluids, and the average time to first fluids was 1.6 h. Lower age, lower triage category and the presence of more severe clinical signs of sepsis increased the probability of receiving fluids and reduced the time to first fluids. Recognising these patient and non-patient related factors will assist in designing and implementing quality performance improvement initiatives. Awareness of these factors can influence clinician decision making in ED practice environment. Any improvement in sepsis care practice will result in mortality benefits for patients. However, other factors we haven’t explored may determine why and when sepsis patients receive fluids in the ED. Further studies are required to establish a comprehensive understanding of the subjective factors associated with patients receiving early intravenous fluid administration in the emergency department.

## Data Availability

The data analysed in this study are not publicly available due to ethical restrictions.

## Abbreviations

AIC: Akaike Information Criterion
AUC: Area Under the Receiver Operator Curve
ED: Emergency Department
eMR: Electronic Medical Records
mSOFA: Modified Sequential Organ Failure Assessment
